# Innovative Nutrition Strategies for Chronic Disease Prevention: Insights from Research to Real-World Impact

**DOI:** 10.3390/nu17121986

**Published:** 2025-06-12

**Authors:** Yingting Cao, George Moschonis

**Affiliations:** 1 Department of Sport, Exercise and Nutrition Sciences, School of Allied Health, Human Services and Sport, La Trobe University, Bundoora, VIC 3086, Australia; tina.cao@latrobe.edu.au; 2 La Trobe Institute for Sustainable Agriculture & Food (LISAF), La Trobe University, Bundoora, VIC 3086, Australia

Chronic diseases, such as cardiovascular disease (CVD), diabetes, certain types of cancer, osteoporosis, and others, are no longer confined to high-income countries; they have become the leading cause of morbidity and mortality worldwide. Despite the clear role of diet as both a risk factor and a preventive measure, scalable and evidence-based nutrition interventions remain underutilized. The Special Issue “*Innovative Strategies for Preventing Nutrition-Related Chronic Diseases*” addresses this gap by showcasing high-quality original research studies and reviews that explore emerging, multi-level, and interdisciplinary strategies in nutrition science.

A total of six contributions were included in the Special Issue: two randomized controlled trials, one qualitative study, two reviews, and one study protocol. Together, these articles span diverse methodologies and target populations and underscore the importance of translational research and innovations in shaping the future of chronic disease prevention.

## 1. Objective Biomarkers for Dietary Assessment

In the field of dietary intake monitoring and nutritional biomarker discovery, Clarke et al. [1] conducted a secondary data analysis of a single-arm dietary intervention in which Australian adults with overweight or obesity followed a high-fruit-and-vegetable (F&V) diet for 10 weeks. Using urinary metabolite profiles as indicators of dietary adherence and machine learning approaches, the study identified 21 significant urinary metabolites, 11 of which showed changes as early as week 2, providing promising non-invasive biomarkers of dietary intake. These included acetic acid, choline, fumaric acid, and L-tyrosine, among others. The study demonstrates how metabolite profiling can offer a more objective and scalable method for dietary intake monitoring in clinical and population-level interventions.

## 2. Probiotics as Adjuncts in Chronic Disease Management

Probiotic supplementation emerged as a prominent theme, with two studies targeting different at-risk groups. Zikou et al. [2] conducted a 6-month, double-blind, placebo-controlled trial in Greek adults with type 2 diabetes, demonstrating that a multi-strain probiotic supplement led to significantly higher reductions in HbA1c, fasting glucose, and total cholesterol compared to the placebo group. Importantly, these effects remained robust even after adjusting for changes in waist circumference, underscoring a potentially direct metabolic benefit of the examined probiotic supplement on the glycemic profile of people with type 2 diabetes.

Complementing this, Resciniti et al. [3] introduced a study protocol for a 12-month randomized controlled trial in early postmenopausal women in Australia. This trial evaluates a Lactobacilli-based supplement’s effects on volumetric and areal bone mineral density, inflammatory markers, vitamin K2 levels, and gut microbiota composition. The study is based on growing evidence linking gut microbiota to bone health, aiming to clarify the role of probiotics in osteoporosis prevention.

## 3. Systems-Level Strategies and Implementation Frameworks

Considering that individual behavior changes occur within the context of broader systems, Stotz et al. [4] contributed a qualitative, stakeholder-informed adaptation of an existing Theory of Change (ToC) for the Gus Schumacher Produce Prescription Program. Through interviews with healthcare providers, food security experts, and program evaluators, the study identified mechanisms linking produce prescriptions to improved dietary intake, healthcare engagement, and equity. This adapted ToC offers a model for scaling similar interventions in other contexts while embedding equity and cross-sectoral collaboration as core elements.

## 4. Technology and Mechanistic Insights

The integration of advanced technologies into nutrition practice was reviewed by Kassem et al. [5], who performed a narrative review and provided a comprehensive synthesis of evidence on the role of artificial intelligence (AI) in dietary assessment, personalized nutrition, and public health surveillance. The authors discussed tools such as image-based food recognition systems, mobile apps, and predictive algorithms, while also acknowledging limitations related to algorithmic bias, data quality, and regulatory frameworks. As AI becomes further integrated into nutrition care, evidence-based validation remains critical.

At the biochemical and mechanistic levels, Tanase et al. [6] contributed a narrative review on the dual role of branched-chain amino acids (BCAAs) in CVD. While elevated BCAA levels are associated with insulin resistance and pro-inflammatory signaling through the mTOR and NF-κB pathways, under certain conditions they may support muscle mass maintenance and metabolic function. The authors call for additional mechanistic studies to clarify these effects and assess their potential as biomarkers or therapeutic targets.

## 5. Concluding Remarks

Collectively, the studies in this Special Issue reflect the breadth and depth of innovative nutrition science aiming to address chronic disease prevention from multiple angles, including molecular, behavioral, technological, and systemic. From biomarker development and mechanistic exploration to AI-driven tools and stakeholder-informed frameworks, the studies collectively illustrate a forward-thinking, integrative approach to nutrition research.

Despite the inherent complexity of translating these findings across diverse cultural, clinical, and socioeconomic contexts, these contributions reflect the promise of interdisciplinary, evidence-informed strategies. Whether through personalized microbiome-targeted approaches, system-level innovations such as produce prescriptions, or emerging AI solutions, nutrition science is becoming increasingly aligned with real-world application and population health needs.

We thank all the contributing authors and reviewers whose work has shaped this timely collection. We hope this Special Issue inspires further research and implementation efforts that can make meaningful strides in reducing the global burden of chronic diseases through innovations in nutrition.
